# Serum Bile Acids Profiling in Inflammatory Bowel Disease Patients Treated with Anti-TNFs

**DOI:** 10.3390/cells8080817

**Published:** 2019-08-02

**Authors:** Giulia Roda, Emanuele Porru, Konstantinos Katsanos, Alexandros Skamnelos, Kallirroi Kyriakidi, Gionata Fiorino, Dimitrios Christodoulou, Silvio Danese, Aldo Roda

**Affiliations:** 1 IBD Unit, Humanitas Research Hospital, 20089 Milan, Italy; 2 Department of Chemistry G. Ciamician, Alma Mater Studiorum University of Bologna, 40100 Bologna, Italy; 3 Division of Gastroenterology, Department of Internal Medicine, Faculty of Medicine, University of Ioannina School of Health Sciences, 45110 Ioannina, Greece; 4 Department of Biomedical Sciences, Humanitas University, Pieve Emanuele, 20089 Milan, Italy

**Keywords:** Crohn’s disease, ulcerative colitis, absorption, bile acids, secondary bile acids

## Abstract

Background: Inflammatory bowel diseases (IBD), ulcerative colitis (UC), and Crohn’s disease (CD), represent systematic chronic conditions with a deficient intestinal absorption. We first attempt to investigate the serum bile acids (sBAs) profile in a large cohort of IBD patients to evaluate changes under anti-TNF alpha treatment. Methods: Forty CD and 40 UC patients were enrolled and BAs were quantified by high-pressure liquid chromatography-electrospray-tandem mass spectrometry (HPLC-ES-MS/MS). Up to 15 different sBAs concentrations and clinical biomarkers where added to a Principal Component Analysis (PCA) to discriminate IBD from healthy conditions and treatment. Results: PCA allowed a separation into two clusters within CD (biologic-free patients and patients treated with anti-TNF alpha drugs and healthy subjects) but not UC. The first included CD. CD patients receiving anti-TNF alpha have an increase in total sBAs (4.11 ± 1.23 μM) compared to patients not exposed. Secondary BAs significantly increase after anti-TNF alpha treatment (1.54 ± 0.83 μM). Furthermore, multivariate analysis based on sBA concentration highlighted a different qualitative sBAs profile for UC and CD patients treated with conventional therapy. Conclusion: According to our results, anti-TNF alpha in CD restores the sBA profile by re-establishing the physiological levels. These findings indicate that, secondary BAs might serve as an indirect biomarker of the healing process.

## 1. Introduction

Inflammatory bowel diseases (IBD), which include ulcerative colitis (UC) and Crohn’s disease (CD), represent chronic inflammatory conditions with a deficient intestinal absorption as well as an impaired hepatic spill over. These alterations lead to non-physiological concentration of bile acids (BAs) in peripheral blood, bile, and intestinal content [1]. Moreover, parameters like circadian rhythm [2], post-prandial peaks [3], gallbladder and intestinal motility and inflammation [4] as well as hepatic uptake and secretion [5] can affect the levels of sBAs.

BAs are endogenous acidic steroids synthetized in the liver from cholesterol, representing its main metabolites and the main excretion pathway from the body [6]. BAs are known for their role in lipid transport through enterohepatic circulation, but recently the understanding of their function as agonists of Farnesoid X receptor (FXR) [7] and TGR5 [8] has unlighted other physiological functions, in which they act as enteric hormones. Several studies have evaluated BAs metabolism in relation to hepatobiliary and intestinal diseases [9,10] but often limited accurate selection of patient population, sample size and analytical methods are critical and limiting factors [11,12].

The enterohepatic circulation is a complex mechanism which permits the amount of BAs in the body to be kept constant, hampering an excessive fecal loss, and to regulate the gut microbiota composition, strictly involved in the health and disease of the individual [13]. BAs are mainly reabsorbed in the terminal ileum through an active transport mechanism selective for their conjugates with glycine and taurine and inefficient for unconjugated BAs. Passive absorption is also involved and limited to unconjugated BAs particularly for dihydroxy BAs such as chenodeoxycholic and deoxycholic acid with higher lipophilicity in respect to trihydroxylated cholic acid. The unabsorbed fraction undergoes deconjugation and 7-α-dehydroxylation in the colon by gut microbiota, allowing partial passive absorption and maintenance of the physiological pool size [14]. In CD, intestinal inflammation, often associated to a deficit of active transporters in the ileum, is responsible for a BAs malabsorption (BAM) and consequently for both an increased fecal loss and a reduction of their physiological pool [15]. In UC, the involvement of the large intestine might account for a deficit in the passive absorption occurring in the colonic tract, with consequent variation in the BAs pool as well. Moreover, the altered microbiota in these patients could impact and modify the BAs qualitative composition which in the enterohepatic and systemic circulation. The microbiota acts on deconjugation of glycine and taurine conjugated and in the 7-dehydroxylation of unconjugated BAs. This is particularly relevant when some of them specifically act as enteric hormones controlling FXR or TGR5 receptors [16]. Although it is clear that BAs enterohepatic circulation is affected in IBD, only few and disagreeing studies report the alteration in their serum or fecal concentrations and their relationship with different treatments [17]. This study represents the first attempt to screenshot serum BAs profiles in a large cohort of IBD patients to evaluate the effect and efficacy of anti-TNF alpha treatment on BAs metabolism. Understanding whether specific BAs profiles are associated to disease phenotype, disease progression or treatment might help to introduce them as a toolkit in clinical practice to assess disease states, severity, and response to treatment like mucosal healing as a companion diagnostic.

## 2. Materials and Methods

### 2.1. Study Population

All consecutive patients seen between May 31, 2018 and July 1 2018 in one referral center in Greece were prospectively enrolled. Patients could be included if their age was greater than 18 years old, a confirmed diagnosis of IBD by conventional endoscopic and histological criteria, absence of concomitant liver diseases. Patients with a personal history of colectomy, a diagnosis of secondary sclerosing cholangitis, liver dysfunction or a history of orthotopic liver transplant (OLT) were excluded.

For all patients clinical and demographic information, including sex, age, Montreal classification, age at diagnosis, concomitant treatments (5-ASA, steroids, immunosuppressant drugs), endoscopy disease activity, and clinical disease activity, were collected. Laboratory tests excluding liver involvement (total and direct bilirubin, aspartate aminotransferase (AST), alanine transferase (ALT)) and disease activity (platelets (PLT) and C reactive protein) were collected.

Clinical activity was scored according to the Mayo score for UC [18], and the Harvey–Bradshaw index for CD [19]. Endoscopic activity was evaluated according to the Mayo endoscopic score for UC and the Simple Endoscopic Score (SES) for CD [18,20].

A group of 20 healthy subjects (CTRL) was introduced to obtain reference values for sBAs levels. The subjects were under regular diet and none of them had history of alimentary disorders, intestinal diseases or hepatic dysfunction.

A fasting serum sample was obtained from each patient and then stored at −20 °C before sBAs analysis.

### 2.2. Study Outcomes

The primary outcome of this study was to assess BAs profile in patients with IBD, both UC and CD, in comparison to healthy subjects.

The secondary outcomes were to assess BAs profile in response to different regimens treatment (anti-TNFs, steroids, conventional therapy like 5-ASA); to assess BAs profile based on disease extension, disease duration, age at diagnosis, and inflammatory state.

### 2.3. sBA Analysis

Pure standards of all the studied BAs, namely cholic acid (CA), chenodeoxycholic acid (CDCA), deoxycholic acid (DCA), ursodeoxycholic acid (UDCA), lithocholic acid (LCA), and their respective glycine and taurine conjugated, were purchased from Sigma-Aldrich (ST. Louis, MO, USA). All the studied BAs were identified and quantified by high-pressure liquid chromatography-electrospray-tandem mass spectrometry (HPLC-ES-MS/MS) using a previously validated method [21] suitable for BAs determination in plasma or serum after appropriate clean-up of pre-analytical procedures. Liquid chromatography analysis was performed using an Alliance HPLC system model 2695 from Waters combined with a triple quadruple mass spectrometer QUATTRO-LC (Micromass; Waters) using an electrospray interface. BAs were separated by elution gradient mode with a mobile phase composed of a mixture ammonium acetate buffer 15 mM, pH 8.0 (Solvent A) and acetonitrile: methanol = 75:25 v/v (Solvent B). All the chromatograms were acquired in electrospray negative ionization with the mass spectrometer operating in multiple reactions monitoring mode. Up to 15 BAs where identified and quantified in plasma with a limit of quantification suitable for their accurate analysis even in patients with low BAs concentration.

### 2.4. Ethical Considerations

This study was performed according to the directive of the Greek National Committee for Data Protection (HDPA 2472/1997) and was approved by the local ethics committee (n.12/10-5-2018). All patients signed an informed consent form.

### 2.5. Statistical Analysis

To visualize the clustering of the different groups of patients, PCA was performed with Unscrambler X version 10.4 (CAMO Software, Oslo, Norway). Parameters used for the multivariate analysis were the determined concentrations of the following different classes: free SBAs, glycine-conjugate and taurine-conjugate sBAs (five BAs for each group), primary and secondary BAs, primary and secondary ratio. Further PCAs were carried out using single BA concentrations to attempt to evaluate which sBAs are altered in IBD and in patients treated with anti-TNFα therapy.

In addition, patient ages, disease duration, and clinical biomarkers were used for chemometric analysis and interpretation of the results. Total bilirubin (TBIL), direct bilirubin (DBIL), ALT, (ALT, endoscopic activity, clinical disease activity, and PLT, C-reactive protein (CRP) were collected.

Data were pre-processed converting concentration to log (concentration) and using the “autoscale” function of the software.

Univariable statistical data analysis was performed using the total serum BA (TSBA) and other sBA concentrations in the different groups using GraphPad Prism 5.0 software (La Jolla, CA, USA). The quantitative data are presented as mean ± standard deviation (SD). Discriminative marker variables were determined based on the absolute loadings and the variances explained by the PCs. A paired t-test was used to assess specific differences in the SBA quali-quantitative concentration between the different groups. The comparisons between healthy, treated and untreated patients were performed setting the level of statistical significance at *p* value <0.05.

Hotelling T2 and Q were used as statistic methods to detect possible outliers. The confidence interval was settled at 95%. The T^2^ and Q residuals do not indicate outliers for the reported PCAs.

## 3. Results

### 3.1. Demographic and Clinical Characteristics

Eighty patients with IBD, 40 CD, and 40 UC patients were prospectively enrolled. Fifty percent of patients in each group were in treatment with biologics drugs such as Golimumab (Simponi, Janssen Biologics), Adalimumab (Humira, Abbvie), Infliximab (Remicade, Schering-Plough). The remaining 50% of patients had never received anti-TNF alpha treatment in their disease history. The demographic and clinical characteristics of the enrolled patients are described in Table 1. Liver function tests were within the normal range in all the samples where data were available. No patient had a prior history of abdominal, liver surgery and OLT.

### 3.2. Principal Component Analysis

**Figure 1:**
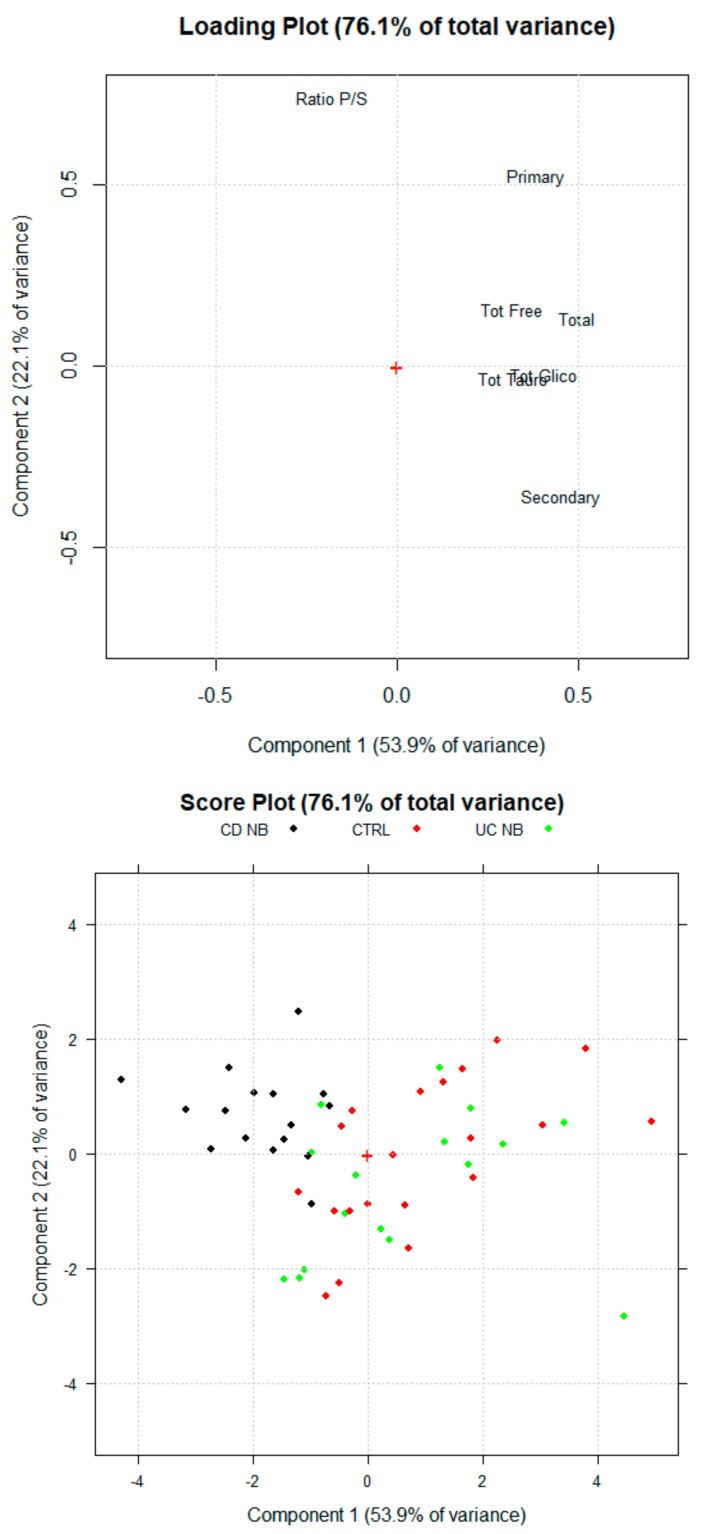
Scores Plot and Loading Plot of the PCA (principal component analysis) for healthy subjects (CTR, Ulcerative colitis (UC NB) and Crohn’s disease (CD NB) patients UC treated with conventional therapies. The first PCA explain the 53.9% of the total variance among the samples. The second PC explain the 22.1% of the total variance.

First, multivariate analysis was carried out by considering concentrations of the different sBAs classes as selected variables to discriminate CTRL subjects, UC and CD patients in conventional treatments. The outputs of the PCA are reported in Figure 1. sBA profiles are similar between UC patients and healthy subjects. The loading plots show that higher levels of sBA correlate with UC patients (sBAs 3.74 ± 1.44 uM) and CTRL subjects (sBAs 3.94 ± 2.12 μM) while lower levels are associated to CD patients (2.25 ± 1.45 μM). Nevertheless, the loading plot highlights how the concentrations of the BA classes are important for a correct discrimination between CD and UC patients. CD patients show higher levels of the ratio between primary and secondary sBAs.

### 3.3. Serum BA Profile in CD Patients Treated with Anti-TNF Alpha

**Figure 2:**
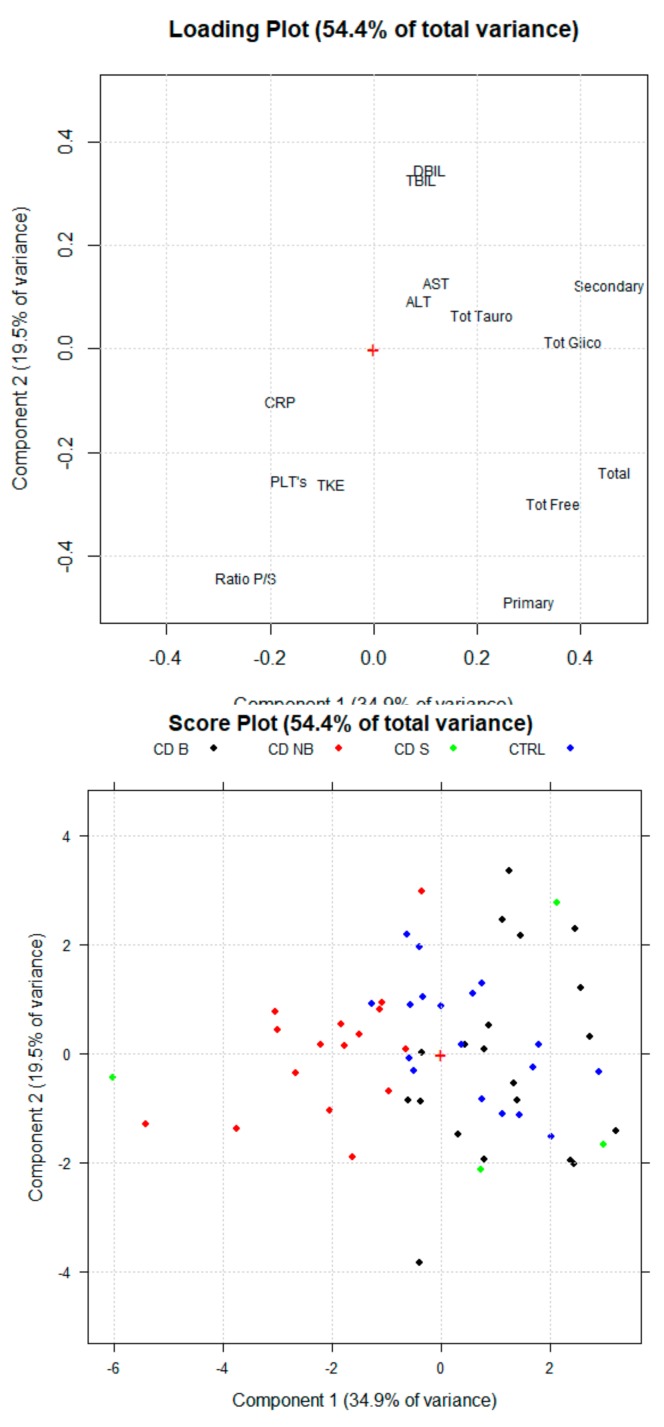
Principal component analysis (PCA) scores Plot and Loading Plot for healthy subjects (CTRL), Crohn’s disease patients treated with anti-TNF alpha (CD B) and Crohn’s disease patients treated with conventional therapy (CD NB). The first PC explains 34.9% of the total variance among the samples. CD B and CRTL clusters are on the positive side of the PC1, while CD NB cluster is on the negative side of the PC1. The second PC explain 19.5% of the total variance, even though clustering was not assessed for groups. Crohn’s disease patients with steroid treatment (CD S) is plotted.

**Figure 3:**
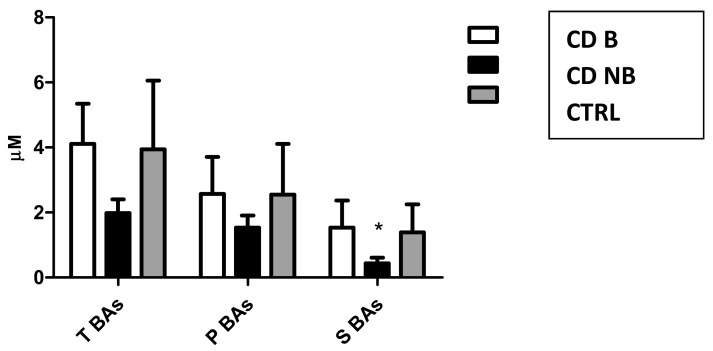
Mean concentrations of total (TSBAs), primary (PBAs) and secondary bile acids (SBAs) in Crohn’s disease patients treated with anti-TNF alpha (CD B), Crohn’s disease patients treated with conventional treatment (CD NB) and healthy subjects (CTRL).

A first PCA was carried out using concentrations of the different BA classes and biomarkers as indicated in the method section. The multivariate analysis technique highlighted a different sBA qualitative profile between CTRL, patients treated with anti-TNF alpha (CD B) and patients treated with conventional therapy (CD NB) (Figure 2). Indeed, the new orthogonal space obtained by PCA demonstrates a quite clear separation on the first principal component (PC1). According to the PCA, higher levels of sBA classes are associated with CTRL (sBAs 3.94 ± 2.12 μM) and CD B patients (sBAs 4.11 ± 1.23 μM), while lower levels have been associated with CD NB patients (sBAs 1.98 ± 0.42 μM). Of note, secondary BA concentrations are the most discriminating parameter contributing to the clustering. Secondary BAs significantly increase (*p* < 0.05) after anti-TNF alpha (1.54 ± 0.83 μM) compared to CD NB (0.44 ± 0.17 μM). CD B patients reach secondary BA levels similar to the CTRL group (1.39 ± 0.86 μM). Indeed, the loading plot of the multivariate analysis describes the relative weight of each variable in the clustering of the samples on each PC. Secondary BAs and total sBAs have highest loading values for the PC1, highlighting the correlation between these variables and the clusters of CD B patients and the CTRL group. On the other hand, the lowest loading value for the PC1 of the mean ratio between primary and secondary sBAs is associated with the cluster of the CD NB patients. The mean ratio between primary and secondary sBAs decreases (*p* < 0.05) in CD NB patients (2.25 ± 1.45 μM) compared to CD B (4.00 ± 1.87 μM), reaching values similar to those of CTRL group (1.93 ± 0.95) (Figure 3).

**Figure 4:**
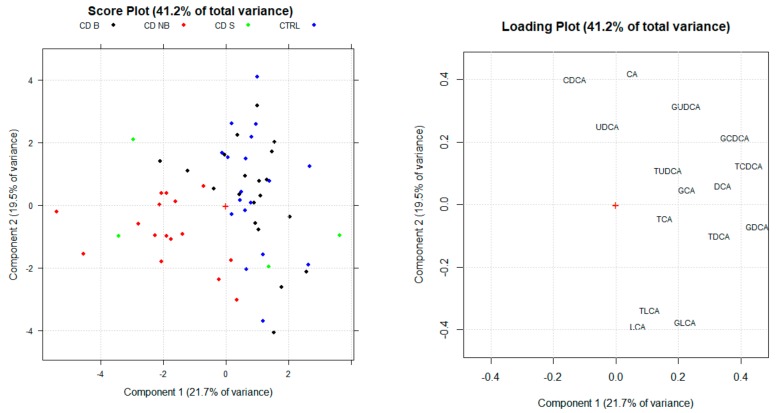
Score plot and loading plot of the Principal component analysis (PCA) obtained by considering single BA concentrations as selected variables. PC1 and PC2 explain the 41.2% of the total variance.

A second multivariate analysis was carried out using only single sBA concentrations to investigate which ones are mainly characterizing for the studied groups. The output of the second PCAs are reported in Figure 4. As expected, high serum concentrations of the main secondary sBAs (deoxycholic acid and its conjugated forms) are associated with CD B and CTRL clusters. Therefore, high plasma levels of other BAs as chenodeoxycholic acid and its conjugated forms are also correlated with CD B and CTRL subject. Specifically, according with the loading value on the first PC, the taurine and glycine conjugated forms seem to be the main variables for discrimination between groups.

Three of the CD NB patients (patients receiving steroid treatment) were clustered in the CD B along the positive side of PC1. Steroid therapy has been reported to restore BA plasma concentration [22]. Consequently, these patients have been considered as a separate group.

### 3.4. sBA Profile in UC Patients Treated with Anti-TNF Alpha Therapy

**Figure 5:**
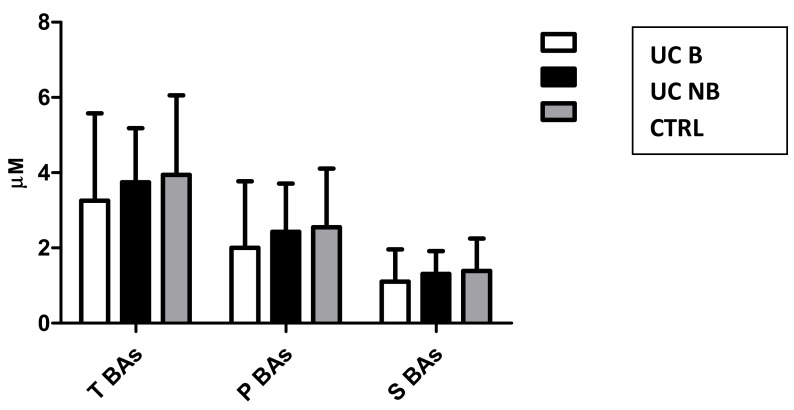
Mean concentrations of total (T BAs), primary (P BAs), and secondary (S BAs) BAs in ulcerative colitis patients treated with anti-TNF alpha (UD B), ulcerative colitis patients treated with conventional therapy (UC NB), and healthy subjects (CTRL).

**Figure 6:**
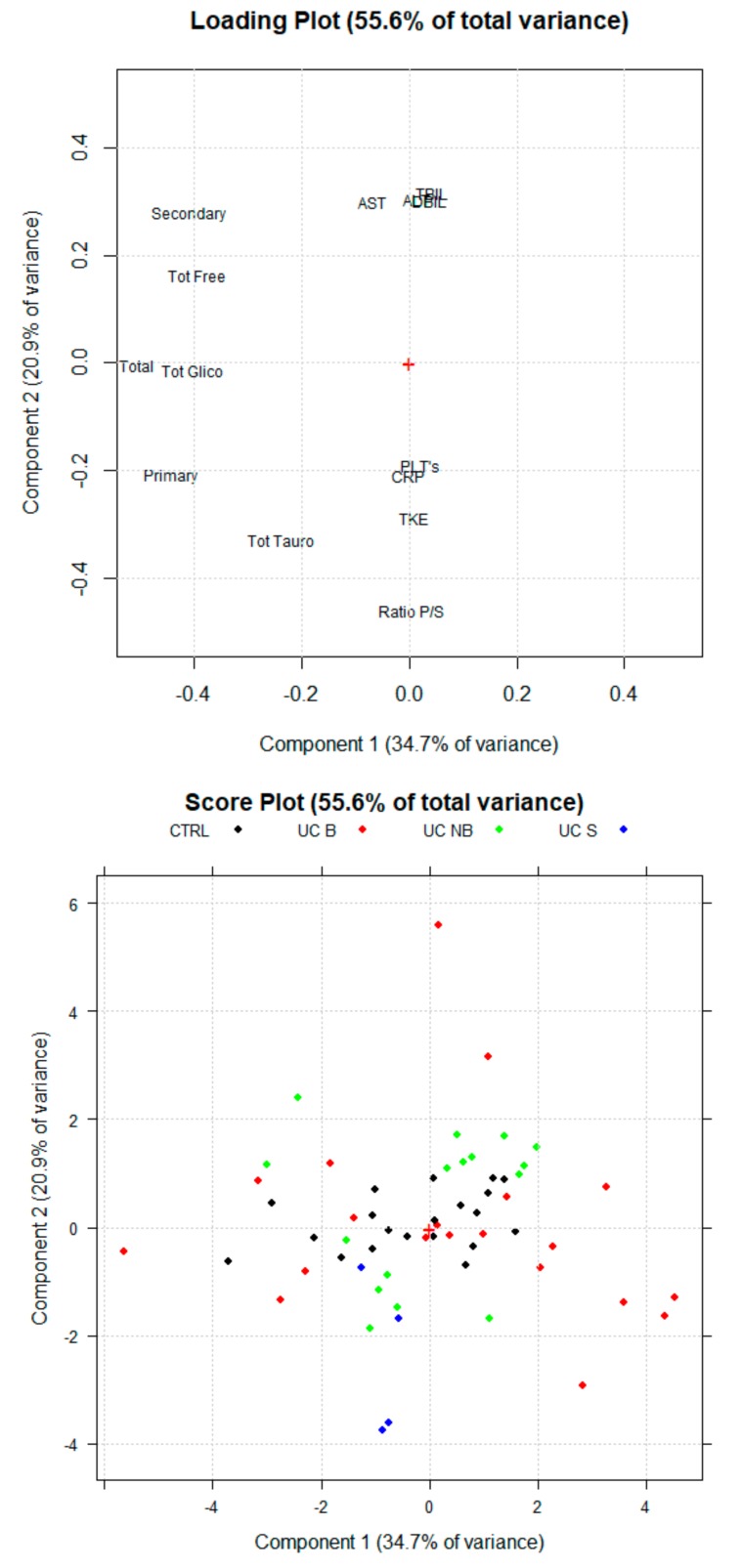
Scores Plot and Loading Plot of the Principal Component analysis (PCA) for healthy subjects (CTRL), ulcerative colitis patients treated with anti-TNF alpha (UC B) and ulcerative colitis patients treated with conventional therapy (UC NB) groups. The first PC explain 25% of the total variance among the samples. The second PC explain 19.5% of the total variance.

Mean sBAs concentrations in patients treated with anti-TNF alpha (UC B), conventional therapy (UC NB), and CTRL were respectively 3.26 ± 2.32, 3.74 ± 1.44, and 3.94 ± 2.12 μM (p value n.s.) (Figure 5). PCA was carried out using concentrations of the different BA classes and biomarkers as indicated in the method section. No differences were assessed between groups (p value ns) (Figure 6). Different profile between groups was not assessed also when single sBAs were used for further PCAs. Indeed, no specific BA showed significant concentration variations over the treatment.

### 3.5. Secondary Outcomes

#### 3.5.1. Disease Duration

Median disease duration for CD patients was 12 years (IQR 5.75-15.5), while for UC patients 13 years (IQR 5-21). Serum BAs were analyzed by dichotomizing and comparing within the CD B and CD NB patients with time to diagnosis less than 12 years with patients with more than 12 years and the same in UC B.

The values of sBAs in CD B and CD NB patients with less than 12 years disease duration were respectively 4.11 ± 1.41 and 2.07 ± 0.36 μM, while in patients with longer disease they were respectively 4.19 ± 1.04 and 1.86 ± 0.41 μM. sBA levels were not significantly different (p value < 0.05) between groups with the same treatment and disease duration. The same results were obtained in UC without significant differences between groups. Indeed, concentrations of sBAs in UC B and UC NB patients with longer disease were respectively 3.49 ± 2.23 and 3.58 ± 0.89 μM, while in patients with shorter disease were respectively 2.88 ± 2.26 and 3.95 ± 1.58 μM.

#### 3.5.2. Age at Diagnosis

Median age at diagnosis was 30 (IQR 24–51) in the CD group and 37 (IQR 40–72) in the UC group. SBAs were analyzed by dichotomizing based on median age. The values of sBAs in CD B and CD NB patients with earlier diagnosis were respectively 4.07 ± 1.31 and 1.99 ± 0.41 μM, while in older patients at diagnosis they were respectively 4.34 ± 0.92 and 1.90 ± 0.50 μM. SBA levels were not significantly different (*p* value < 0.05) between the groups with the same pharmacological treatment and no specific BA was associated with the age at diagnosis or biological treatment. No significant difference was determined in UC patients, where the sBAs levels in UC B and UC NB patients with earlier diagnosis were respectively 3.23 ± 2.59 and 3.95 ± 1.58 μM, while in older patients at diagnosis they were 3.18 ± 1.99 and 3.53 ±1.24 μM.

#### 3.5.3. Disease Extension

With respect to disease location and according to the Montreal classification, the majority of the UC B and UC NB were classified as E3 (75% E3 and 25% E2). Among CD B patients, 11 out of 20 were L1B1, 1 was L1B2 and two were L1B3. Only one out of 20 CD B patients was L2B3, while two were L3B2 and three were L3B1. Eight out of 20 CD NB were L1B1, one L1B2, three were L2B1, one was L3B3, three were L3B1 and one L4B1. For three patients location was not available. sBAs qualitative composition was analyzed by dichotomizing and basing on the disease extension between patients with inflammation limited to ileum (L1) and patient with more extensive illness (L2 and, L3). The values of sBAs in CD B and CD NB patients with L1 classifications were respectively 4.25 ± 1.38 and 1.94 ± 0.30 μM. The levels of sBAs in CD B and CD NB patients with L2 classification were respectively 2.93 and 1.95 ± 0.14 μM. This trend is respected also in patients with L3 classification, where the levels of TSBAs in CD B and CD NB patients were respectively 3.77 ± 0.77 μM and 2.06 ± 0.53 μM. L1 patients showed the greatest concentration in relation to ileal inflammation which, according to the literature [23], affects the active transport of BAs. Despite these results, the increase in sBAs levels after biological treatment is met even in L2 and L3 patients without a specific localization of the illness in the ileum. As reported above, the increase in sBAs levels is strictly correlated with secondary BAs. Specifically, DCA reaches concentrations after biological treatment in L1, L2, and L3 patients respectively of 0.61 ± 0.41 μM, 0.33 μM and 0.83 ± 0.68 μM without significant differences if compared with DCA levels of the CTRL subjects (0.56 ± 0.40 μM). On the other hand, patients under conventional therapy reach DCA concentrations of 0.07 ± 0.07 μM, 0.04 ± 0.04 μM and 0.05 ± 0.04 μM respectively in L1, L2, and L3 patients, with significant differences if compared with other groups (*p* < 0.05).

#### 3.5.4. Inflammatory State

SBAs were analyzed by dichotomizing patients treated or not with anti-TNF alpha therapy based on CRP levels using a cut-off of 7. sBAs were respectively 4.25 ± 1.42 μM and 2.03 ± 0.26 μM in CD B and CD NB patients with CRP lower than 7. The levels of sBAs in CD B and CD NB patients with CRP higher than 7 mg/L were respectively 3.79 ± 0.91 μM and 1.95 ± 0.50 μM. This data is consistent with the previously reported data comparing biological and conventional therapy.

SBAs concentrations were assessed within CD B patients with higher or lower CRP values (3.66 ± 0.77 μM and 4.30 ± 1.38 μM respectively). Significant differences (*p* value < 0.05) were determined if secondary sBAs levels (1.03 ±0.54 μM and 1.75 ± 0.85 μM respectively) were considered. According to these results, production and absorption of secondary BAs seem to be affected by the inflammatory state. Biological therapy is more effective in restoring BAs pool when inflammatory response has been quietened by anti-TNF alpha (CRP < 7 mg/L). No significant difference was determined in UC patients with different CRP levels.

#### 3.5.5. Steroid Treatment

The subgroup of 5 CD patients treated with steroid therapy showed higher sBAs pool (3.99 ± 0.80 μM) compared to CD NB (1.98 ± 0.42 μM), reaching concentrations similar to healthy individuals (3.94 ± 2.12 μM). Specifically, steroid treatment improves secondary BAs concentration as well as biological treatment.

## 4. Conclusions

This study represents the first attempt to identify sBAs profiles in IBD patients to evaluate the effect of anti-TNF alpha treatment on their serum profiles.

Qualitative and quantitative variation of sBAs levels might be related to many causes, such as impaired biosynthesis from cholesterol, faulty transport of hepatocytes or enterocytes through the cellular membrane, defective transport among the physiological compartments involved in the enterohepatic circulation, inflammatory state or abnormal bacterial overgrowth in the large intestine. Few studies have analyzed sBAs profiles in IBD patients in specific circumstances [17].

In this study, in order to exclude all the possible misleading variables, we performed a high accurate selection of the patient population, based on several criteria (exclusion of liver involvement by anamnesis and blood tests, exclusion of combination therapy, inclusion of ileum involvement in CD group, extensive colitis in UC group).

According to our results, the total concentration of secondary BAs in serum of CD patients represents the most discriminating parameter. CD patients receiving biologic treatment seems to be able to increase and restore secondary sBA levels similar to those of the healthy subjects compared to CD patients treated with conventional therapy. This result indirectly suggests the positive effect of the treatment on the intestine wall and biofilm microbiota, responsible for primary BAs biotransformation to secondary BAs via 7-dehydroxylation and, in addition for BAs efficient absorption.

Based on our results, disease extension did not affect significantly secondary or sBAs concentration in CD B patients. However, a greater tendency was assessed toward an increase of secondary BAs in patients with ileal disease where the active transport of BAs is affected [23].

Significant differences in secondary BAs levels were found in CD B patients with different CRP values suggesting that TNFs treatment might restores BAs pool depending on the inflammatory state.

Our study for the first time suggests that biological treatment in CD patients restores BAs physiological levels. Particularly, the improvement of DCA and other secondary BA concentrations seems closely associated with the anti-TNFα therapy. These results suggest that the passive absorption in the colon tract of the most lipophilic BAs have been re-established. Moreover, we showed that production and absorption of secondary BAs seem to be affected by the inflammatory state. As expected, biological therapy seems to be more effective in restoring sBAs concentrations when the inflammatory response has been stopped by anti-TNF alpha (CRP < 7).

Indirectly this study further highlights the underestimated role of secondary BAs often considered excretory molecules eliminated in stools without any peculiar properties. Secondary BAs and particularly DCA still play a physiological role in controlling the FXR activity in the intestine and further studies are required to better define this role. An excessive production and accumulation of secondary BAs in subjects with bacteria overgrowth was neglected and can be the cause of hepatobiliary diseases. This is related to the potential toxicity of DCA like detergent and lipophilic compound, inducing also an increase in biliary cholesterol secretion leading to cholesterol gallstones formation. An ideal cocktail of primary and secondary BAs is the requisite to keep equilibrated the physiological role of BAs. The BA pool is controlled not only by its hepatic synthesis but mainly by the intestinal wall and bacteria metabolism that is impaired in IBD patients.

One limitation of our study is the sample size especially when specific treatment was considered such as steroids. A large cohort of selected patients will be therefore necessary to fully define the BAs role in IBD. However, generally these data show that each subject presents a peculiar sBA composition and response to treatment that should be more carefully evaluated in terms of time and dose by following the complete sBA profile and if possible the serum level of the administered drug to relate the bioavailability of the drug (pharmacokinetic) with the sBA levels.

Classification models (PLS-DA, SIMCA) in multivariate analysis could be created if more patients were to be enrolled for this kind of study. According to our results, these models could be powerful tools also in clinical practice to obtain important information for preliminary diagnosis, disease activity, and the healing process of IBD patients.

In conclusion, this study suggests restoring the effect of the TNF-alpha therapy on the enterohepatic circulation in CD. In particular, our preliminary results open up new perspectives on the role of sBAs as non-invasive biomarkers for clinical remission in Crohn’s disease and potentially for mucosal healing in ulcerative colitis and colonic Crohn’s disease in relationship to microbiota and bile acids. Therefore, a complete and systematic characterization of the BAs profile, including secondary metabolites, can be of great help in the light of the concept of precision medicine in IBD patients.

## Figures and Tables

**Table 1:** Demographic and clinical characteristics of patients.

	PLT’s (×1000)	CRP (0–6 mg/L)	Endoscopic activity (0 = not active, 1 = mild, 2 = moderate, 3 = severe)	Disease activity (0 = not active, 1 = mild, 2 = moderate, 3 = severe)	TBIL	Montreal Classification	Date of Diagnosis	Medication
CD1	349	9	1	0	0.4	L1B1	2012	infliximab
CD2	303	5	0	0	0.6	L1B3	2011	infliximab
CD3	284	2	2	1	0.4	L2B3	1990	infliximab
CD4	250	2	0	0	0.9	L1B1	2009	infliximab
CD5	406	7	1	1	0.5	L3B1	2011	infliximab, methotrexate
CD6	260	2	1	2	0.9	L1B1	NA	adalumimab
CD7	287	2	0	0	0.4	L1B1	2009	adalumimab, 5-ASΑ
CD8	395	29	2	1	1.2	L1B1	2011	infliximab
CD9	281	2	0	0	0.4	L1B2	2013	infliximab
CD10	228	3	0	0	0.9	L1B1	2003	infliximab
CD11	485	14	2	2	0.5	L1B2	2006	infliximab
CD12	303	3	2	1	0.4	L1B1	2010	infliximab
CD13	302	12	1	2	0.4	L1B1	2000	adalumimab, 5-ASΑ
CD14	244	1	0	0	1.2	L1B1	1997	infliximab
CD15	407	5	1	1	0.6	L3B1	2012	infliximab
CD16	183	2	0	0	0.9	L3B1	NA	infliximab
CD17	162	4	0	0	0.5	L1B1	2012	infliximab, AZA, 5-ASA
CD18	245	1	0	0	0.4	L1B1	2012	infliximab
CD19	270	NA	0	0	1.2	L3B2	1997	adalumimab, 5-ASΑ sus
CD20	385	7	1	3	0.6	L3B2	2014	adalumimab
UC1	NA	NA	NA	NA	NA	E3	2008	infliximab
UC2	NA	NA	NA	NA	NA	E3	2012	golimumab
UC3	157	7	0	0	0.7	E2	NA	golimumab, 5-ASA
UC4	381	21	1	1	0.3	E3	2004	golimumab, steroids
UC5	196	4	0	0	1.2	E2	2016	infliximab, AZA, 5-ASA
UC6	253	3	0	0	0.4	E3	1995	infliximab
UC7	267	2	1	1	0.4	E3	1996	infliximab, 5-ASA
UC8	261	5	0	0	0.4	E3	NA	vedolizumab, AZA, 5-ASA
UC9	343	1	0	0	0.4	E3	2006	golimumab, steroids, 5-ASA
UC10	470	6	1	0	0.4	E3	2000	adalimumab, AZA, 5-ASA, ursodeoxycholic acid
UC11	209	5	1	1	0.3	E3	NA	infliximab
UC12	228	2	1	1	0.5	E3	1985	infliximab, steroids
UC13	176	3	0	0	2.5	E3	1984	infliximab
UC14	203	6	2	3	0.6	E3	2006	infliximab, 5-ASA
UC15	242	3	1	1	0.3	E3	1997	infliximab
UC16	322	5	1	2	0.9	E3	2010	infliximab, Aza adalimumab, 5-ASA
UC17	275	2	1	1	0.6	E2	2013	golimumab, steroids, AZA, 5-ASA
UC18	254	5	2	1	0.5	E2	1994	infliximab
UC19	253	5	1	0	1	E3	NA	vedolizumab, 5-ASA
UC20	232	2	0	0	0.6	E2	NA	adalumimab
CD1	323	1	0	0	0.6	L3B1	2011	AZA
CD2	329	6	0	3	0.3	L1B1	2016	no medication
CD3	222	12	0	0	0.2	L1B1	2016	steroids, 5-ASA
CD4	257	2	0	0	0.4	L1B1	2006	AZA
CD5	NA	NA	0	0	NA	L3B1	2008	AZA
CD6	211	3	0	0	0.8	L3B1	1998	AZA
CD7		NA	0	0	NA	L2B1	1989	No medication
CD8	209	4	0	0	1.3	L1B1	2016	AZA, 5-ASA
CD9	312	3	0	0	0.5	L1B1	1997	AZA, 5-ASA
CD10	335	5	0	1	0.6	L1B2	2006	steroids per os/enema
CD11	279	2	0	0	0.8	L1B1	2010	AZA
CD12	258	7	0	0	0.9	L1B1	2015	AZA, 5-ASA
CD13	409	27	0	0	0.4	L4B1	2017	steroids
CD14	166	6	1	0	1	L2B1	1974	5-ASA, steroids enema
CD15		NA	0	0	NA	L2B1	2013	AZA, 5-ASA
CD16	455	20	2	2	0.3	L3B3	NA	no medication
CD17	326	26	1	0	0.3	L1B1	2002	no medication
CD18	NA	NA	NA	NA	NA	NA	NA	NA
CD19	NA	NA	NA	NA	NA	NA	NA	NA
CD20	NA	NA	NA	NA	NA	NA	NA	NA
UC1	NA	NA	0	0	0.15	E3	1993	methotrexate, 5-ASA
UC2	NA	NA	0	1	1.4	E2	2015	5-ASA, per os/sus
UC3	219	2	0	0	1.3	E3	2007	No medication
UC4	181	3	0	0	0.5	E2	2013	AZA, 5-ASA
UC5	NA	NA	0	0	ΝΑ	E3	2004	5-ASA per os/sus
UC6	457	2	2	1	0.5	E3	1990	5-ASA per os/sus, steroids
UC7	NA	NA	1	0	ΝΑ	E2	2010	5-ASA
UC8	346	5	2	1	0.4	E3	2016	5-ASA
UC9	319	13	0	1	0.5	E2	2016	5-ASA
UC10	224	3	0	0	1	E2	2017	5-ASA per os/sus
UC11	255	2	1	2	0.6	E3	2011	5-ASA
UC12	NA	NA	0	0	NA	E2	2000	NA
UC13	NA	NA	0	0	NA	E3	2009	no medication
UC14	NA	NA	0	0		E3	2017	NA
UC15	242	3	0	0	0.4	E2	2017	AZA, steroids, 5-ASA
UC16	279	5	0	1	0.5	E3	1997	AZA, 5-ASA
UC17	201	NA	0	1	0.4	E3	2007	no medication
UC18	401	7	0	0	0.6	E3	1989	methotrexate, steroids
UC19	199	37	3	3	0.4	E3	2013	5-ASA (oral and topical), steroids
UC20	285	4	1	0	0.5	E2	2007	5-ASA (oral and topical)

PLT: platelets, CRP: C reactive protein, TBIL: total bilirubin, AZA: azathioprine, 5-ASA: 5-aminosalicylic acid.
